# Spermine Promotes the Formation of Conchosporangia in *Pyropia haitanensis* Through Superoxide Anions

**DOI:** 10.3390/md23080309

**Published:** 2025-07-30

**Authors:** Tingting Niu, Haike Qian, Lufan Cheng, Qijun Luo, Juanjuan Chen, Rui Yang, Peng Zhang, Tiegan Wang, Haimin Chen

**Affiliations:** 1 Collaborative Innovation Center for Zhejiang Marine High-Efficiency and Healthy Aquaculture, Ningbo University, Ningbo 315211, China; niutingting@nbu.edu.cn (T.N.); 18268778619@163.com (H.Q.); chenglufan36@gmail.com (L.C.); luoqijun@nbu.edu.cn (Q.L.); chenjuanjuan@nbu.edu.cn (J.C.); yangrui@nbu.edu.cn (R.Y.); 2 Zhejiang Mariculture Research Institute, Wenzhou 325005, China; zhangpeng20011918@163.com (P.Z.); wtg605@163.com (T.W.)

**Keywords:** *Pyropia haitanensis*, conchosporangia, superoxide anions, spermine, redox homeostasis

## Abstract

The transition from conchocelis to conchosporangia in *Pyropia haitanensis* represents a pivotal stage in its life cycle. As a commercially vital red alga, *P. haitanensis* plays a dominant role in global nori production. The transition governing its sporulation efficiency is pivotal for aquaculture success, yet the underlying regulatory mechanisms, especially their integration with metabolic cues such as polyamines, remain poorly understood. This study uncovered a critical role for the polyamine spermine (SPM) in promoting conchosporangial formation, mediated through the signaling activity of superoxide anions (O_2_·^−^). Treatment with SPM markedly elevated O_2_·^−^ levels, an effect that was effectively inhibited by the NADPH oxidase inhibitor diphenyliodonium chloride (DPI), underscoring the role of O_2_·^−^ as a key signaling molecule. Transcriptomic analysis revealed that SPM enhanced photosynthesis, carbon assimilation, and respiratory metabolism, while simultaneously activating antioxidant enzymes, such as superoxide dismutase (SOD), ascorbate peroxidase (APX), and catalase (CAT), to regulate hydrogen peroxide (H_2_O_2_) levels and maintain redox homeostasis. Furthermore, SPM upregulated genes associated with photosynthetic carbon fixation and the C_2_ oxidative photorespiration pathway, supplying the energy and metabolic resources necessary for this developmental transition. These findings suggested that SPM orchestrated O_2_·^−^ signaling, photosynthetic activity, and antioxidant defenses to facilitate the transition from conchocelis to conchosporangia in *P. haitanensis*.

## 1. Introduction

*Pyropia haitanensis* is a commercially significant red alga (Rhodophyta, Bangiaceae) extensively cultivated along the southeastern coast of China, accounting for approximately three-quarters of the country’s nori production. Its life cycle alternates between two distinct stages: the macroscopic blade gametophyte (thallus) and the microscopic filamentous sporophyte (conchocelis) [1,2]. The transition from conchocelis to conchosporangia is a pivotal phase for producing conchospores, which directly influences the quality and yield of gametophytic thalli. In large-scale cultivation of *P. haitanensis*, environmental factors, including light intensity, temperature, and phosphorus levels, are meticulously controlled to enhance conchosporangial development [3,4,5]. Despite these advances, the molecular mechanisms governing this transition remain largely uncharacterized.

Recent studies highlight the involvement of phytohormones like jasmonic acid, ethylene, and indole-3-acetic acid in the reproductive processes of algae [6,7,8]. For instance, the ethylene precursor 1-aminocyclopropane-1-carboxylic acid (ACC) has been shown to induce the transformation of conchocelis into conchosporangia in *P. haitanensis* [2]. Furthermore, polyamines, small nitrogenous compounds such as putrescine (PUT), spermidine (SPD), and spermine (SPM), are increasingly recognized for their roles in algal reproduction. Distinct from higher plants and other red algae, *P. haitanensis* has developed a unique stress-adaptation strategy that integrates S-adenosylmethionine (SAM)-dependent SPD biosynthesis with the glutathione (GSH) antioxidant system, enabling rapid acclimation to intertidal wet–dry fluctuations [9]. Although polyamines have been shown to promote carpospore output and growth in *Gracilaria cornea* (formerly *Hydropuntia cornea*) [10] and to enhance spore release, survival, and germination in *Gracilariopsis lemaneiformis* [11], their potential role in regulating conchosporangial formation in *P. haitanensis* remains uninvestigated, highlighting a compelling direction for future research.

Reactive oxygen species (ROS), including H_2_O_2_ and O_2_·^−^, are pivotal signaling molecules involved in regulating plant growth, development, and stress responses [12]. In *P. haitanensis*, H_2_O_2_ has been identified as a mediator of ACC-induced conchosporangial formation [2]. ROS are predominantly generated by NADPH oxidases (NOXs), which initiate the production of O_2_·^−^, subsequently dismutated into H_2_O_2_ by SOD [13]. Notably, polyamines play a dual role in ROS dynamics: they can generate and regulate ROS through catabolic pathways, maintaining a balance between oxidative stress and cellular signaling [14,15]. While the signaling role of H_2_O_2_ is well-documented, the involvement of O_2_·^−^ in algal development remains less understood. Emerging evidence suggests its significance in key processes, such as cell cycle progression and gametophyte formation, highlighting its potential as a regulatory molecule [16,17,18,19].

This study explored the role of SPM in regulating conchosporangial development in *P. haitanensis*. Our findings revealed O_2_·^−^ as a critical signaling molecule activated by SPM, which facilitated the formation of conchosporangia. Transcriptomic analyses further demonstrated that SPM promoted this developmental transition by enhancing photosynthesis and carbon assimilation and maintaining oxidative–antioxidative balance. These results shed new light on the molecular mechanisms underlying algal reproduction and offered valuable insights for optimizing *P. haitanensis* cultivation strategies.

## 2. Results

### 2.1. Polyamines Enhance Conchosporangial Formation in P. haitanensis

**Figure 1:**
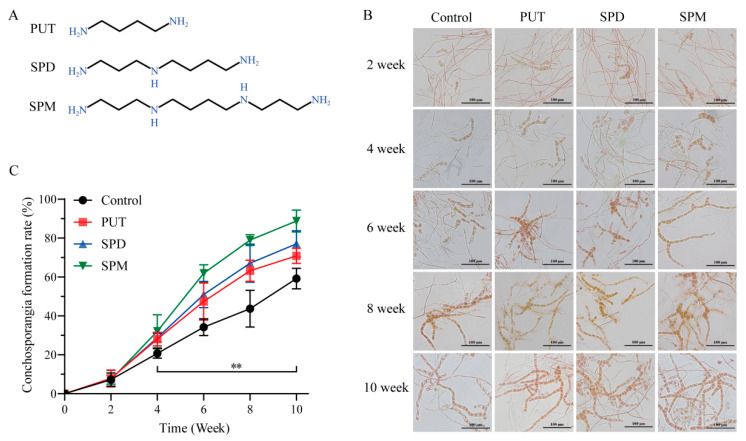
Effect of polyamines on the formation of conchosporangia in *P. haitanensis*. (**A**) Structural formulas for polyamines. (**B**,**C**) Effect of 1 μM polyamines on the formation of conchosporangia and statistics of the proportion of conchosporangial formation. ** *p* < 0.01, compared to the control group (n = 6). Scale bar: 100 μm.

To clarify the involvement of polyamines in conchosporangial development, we first measured the endogenous levels of PUT, SPD, and SPM in *P. haitanensis* (Figure S1). The basal concentrations were quantified as 28.33 μg/g fresh weight (FW) for PUT, 2.09 μg/g FW for SPD, and 10.39 μg/g FW for SPM. To evaluate their physiological roles, exogenous polyamines were administered at concentrations ranging from 1 μM (10^−6^ M) to 100 μM (10^−4^ M). As shown in Figure 1 and Figure S2, following a 2-week treatment period, the emergence of conchosporangia was first observed, with their proportion progressively increasing over time. By the 10th week, the formation rate reached 70–90%, exhibiting a steady upward trend throughout the experimental duration.

Among the polyamines tested, SPM demonstrated the strongest promotive effect on conchosporangial formation. The SPM-treated group achieved a formation rate of 88.78% by the 10th week, the highest observed across all treatments. In comparison, PUT and SPD resulted in relatively lower formation rates. These findings underscored the superior efficacy of SPM in facilitating conchosporangial development. Consequently, SPM was selected for subsequent experiments aimed at elucidating the mechanisms underlying its role in promoting conchosporangial formation.

### 2.2. SPM Enhances Conchosporangial Formation in Both Shell-Living and Free-Living Conchocelis

**Figure 2:**
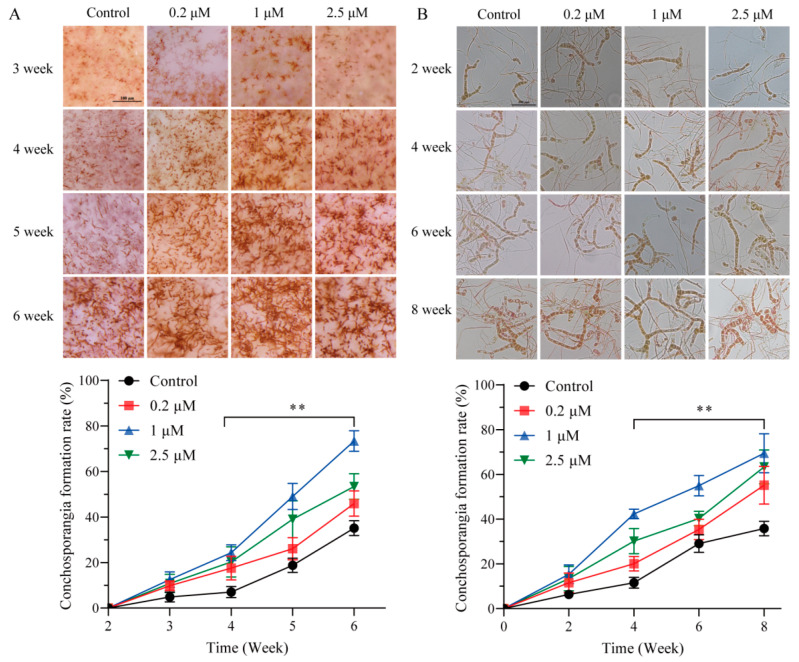
The influence of SPM on the formation of conchosporangia in *P. haitanensis*. Effect of different concentrations of SPM on the formation of shell-living conchosporangia (**A**) and free-living conchosporangia (**B**) in *P. haitanensis*. Statistical significance was calculated by one-way ANOVA. ** *p* < 0.01, compared to the control group (n = 6). Scale bar: 100 μm.

The effects of SPM on conchosporangial formation in shell-living and free-living conchocelis of *P. haitanensis* are presented in Figure 2. In shell-living conchocelis, SPM treatment at concentrations of 0.2, 1, and 2.5 μM significantly stimulated conchosporangial formation compared to the control group. Conchosporangial clusters first appeared on the shell surface by the 3rd week of treatment, and by the 6th week, the surface was almost fully covered. Among the tested concentrations, 1 μM SPM was the most effective, achieving a formation rate of 73.4%, significantly exceeding that of the control group (*p* < 0.05). Although 0.2 μM and 2.5 μM SPM also enhanced conchosporangial formation, their effects were slightly less pronounced than that of 1 μM (*p* < 0.05).

In free-living conchocelis, treatment with 1 μM SPM similarly resulted in significant promotion of conchosporangial formation, reaching a formation rate of 69.49% after 8 weeks. This rate was markedly higher than that observed in the control group (*p* < 0.05), where conchosporangial formation was minimal. While 0.2 μM and 2.5 μM SPM also increased formation rates relative to the control, their effects were not as robust as those of 1 μM.

These findings conclusively demonstrated that SPM effectively promoted conchosporangial formation in both shell-living and free-living conchocelis, with 1 μM SPM being the optimal concentration. Consequently, 1 μM SPM was selected for subsequent experiments to investigate the mechanisms underlying its promotive effects on conchosporangial formation.

### 2.3. Role of O_2_·^−^ in SPM-Induced Conchosporangial Formation

**Figure 3:**
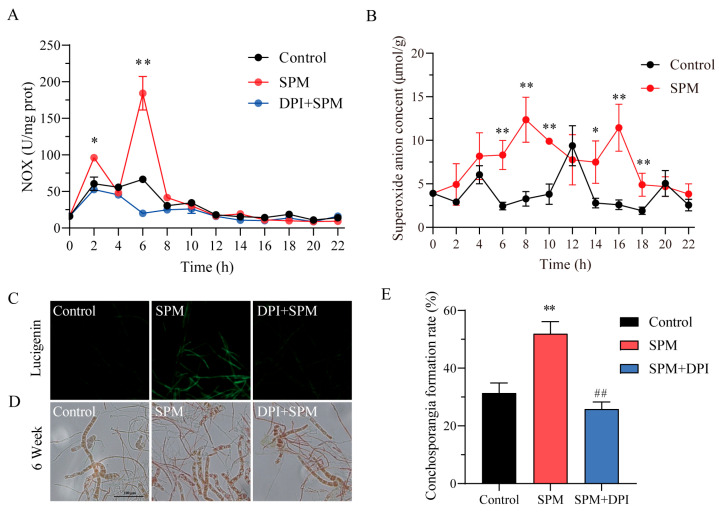
SPM promotes the generation of O_2_·^−^ during conchosporangial formation of *P. haitanensis*. (**A**) NOX enzyme activity. Conchocelis were subjected to maturation conditions and treated with 1 μM SPM. Enzyme activity was assessed at different time points. * *p* < 0.05 and ** *p* < 0.01, compared to the control group (n = 3). (**B**) O_2_·^−^ generation curve. * *p* < 0.05 and ** *p* < 0.01, compared to the control group (n = 3). (**C**) Micrographs of conchocelis stained with lucigenin. Scale bars: 50 μm. Conchocelis was treated with SPM along with NOX inhibitor DPI for 8 h and then stained with lucigenin for 30 min. (**D**) The influence of O_2_·^−^ on conchosporangial formation under maturation conditions for 6 weeks. Scale bars: 100 μm. (**E**) Conchocelis were treated with 1 μM SPM, along with the NOX inhibitor DPI, for 6 weeks. Statistical significance was checked by one-way ANOVA, followed by Tukey’s HSD test. ** *p* < 0.01, compared to the control group (n = 3); ^##^ *p* < 0.01, compared to the SPM group (n = 3).

The role of SPM in generating ROS during conchosporangial formation in *P. haitanensis* was investigated (Figure 3). NOX activity assays exhibited a biphasic response following SPM treatment, with distinct peaks at 2 h (96.12 U/mg protein) and 6 h (184.19 U/mg protein), before gradually returning to baseline by 8 h. Notably, co-treatment with the NOX inhibitor DPI completely suppressed the SPM-induced enhancement of NOX activity (Figure 3A). Consistently, RT-PCR analysis revealed that SPM upregulated the transcript levels of *NOXA* and *NOX5*, consistent with the observed enzyme activity dynamics (Figure S3). To assess the functional impact of NOX activation, O_2_·^−^ and H_2_O_2_ levels were measured in conchocelis exposed to 1 μM SPM over a 24 h period. A significant increase in O_2_·^−^ levels was observed during the first 8 h, reaching a peak concentration of 12.36 μmol/g·wt. A secondary peak (11.43 μmol/g·wt) occurred at 16 h, after which O_2_·^−^ levels gradually returned to baseline (Figure 3B). In contrast, H_2_O_2_ levels remained relatively stable throughout the treatment period (Figure S4), indicating that the observed increase in ROS was primarily attributable to O_2_·^−^ production rather than H_2_O_2_.

Fluorescence microscopy using lucigenin further confirmed the production of O_2_·^−^, as evidenced by strong green fluorescence in conchocelis cells. This fluorescence reached its peak intensity after 8 h of SPM treatment and was notably diminished upon the addition of the DPI, demonstrating the involvement of NOX in O_2_·^−^ generation during this process (Figure 3C).

To establish the role of O_2_·^−^ in SPM-induced conchosporangial formation, conchocelis were treated with 1 μM SPM in the presence of DPI. DPI treatment significantly suppressed conchosporangial formation, reducing the formation rate by 50.2% compared to SPM treatment alone (*p* < 0.05). This marked inhibition underscored the pivotal role of O_2_·^−^ as a signaling molecule in SPM-mediated conchosporangial formation. The reduction in conchosporangial formation correlated strongly with decreased O_2_·^−^ levels, further supporting the hypothesis that O_2_·^−^ served as a critical mediator of this developmental process.

### 2.4. Transcriptomic Profiling of Conchosporangia in P. haitanensis in Response to SPM

To elucidate the molecular mechanisms underlying SPM-induced conchosporangial formation, transcriptome sequencing was performed on *P. haitanensis* conchocelis treated with 1 μM SPM for 24 h. The sequencing generated approximately 2.5 × 10^7^ clean reads with an average length of 150 bp, of which 88.8% were successfully mapped to 11,129 genes in the *P. haitanensis* reference genome. Validation of RNA-seq data through qRT-PCR analysis of 10 randomly selected unigenes demonstrated a high degree of consistency between the transcriptome and qRT-PCR results (Figure S5).

Comparative analysis identified 2310 Differentially expressed genes (DEGs) following SPM treatment, including 1255 upregulated and 1055 downregulated genes (Supplementary Table S1). KEGG pathway enrichment analysis, ranked by significance, revealed that these DEGs were predominantly associated with pathways involved in photosynthetic carbon fixation, photosynthesis, acetate and dicarboxylate metabolism, pyruvate metabolism, carotenoid biosynthesis, alanine, aspartate, and glutamate metabolism, as well as peroxisome functions (Figure S6).

### 2.5. SPM Enhances Photosynthesis in P. haitanensis Conchosporangia

**Figure 4:**
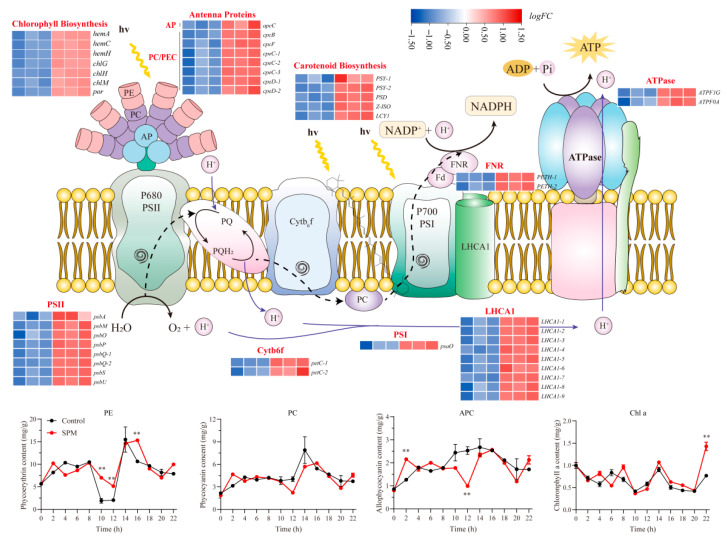
The response of photosynthesis in *P. haitanensis* conchosporangia to SPM. Components highlighted in red represent functional units containing significantly enriched DEGs (|log2FC| > 1, adjusted *p*-value < 0.05). Phycoerythrin (PE), phycocyanin (PC), allophycocyanin (APC), and chlorophyll a content in *P. haitanensis* conchocelis under 1 μM SPM treatment for 24 h. Data represent mean ± SD (*n* = 3). ** *p* < 0.01 vs. control.

SPM treatment significantly impacted the expression of genes related to photosynthesis and photosynthetic carbon fixation pathways (Figure 4). A total of 44 DEGs involved in photosynthesis were identified, all of which were markedly upregulated compared to the control group. Notably, genes encoding components of photosystem I (*psaO*) and photosystem II (*psbA*, *psbM*, *psbO*, *psbP*, *psbQ*, *psbS*, and *psbU*) exhibited substantial upregulation. Similarly, genes associated with the cytochrome b6f complex (*petC*), ferredoxin-NADP^+^ reductase (*petH*), and ATP synthase complex (*atpF*) were significantly upregulated (*p* < 0.05), indicating enhanced light reaction activity.

Additionally, SPM treatment elevated the expression of genes encoding light-harvesting complex proteins (*LHCA*) and phycobiliproteins, including allophycocyanin (*apcC*) and phycocyanin (*cpcB*, *cpcF*, *cpeC*, and *cpeD*). Importantly, this upregulation extended to the major components of the photosynthetic pigment biosynthesis pathway, with key genes involved in chlorophyll a production (*hemA*, *hemC*, *hemH*, *chlG*, *chlH*, *chlM*, and *por*) and carotenoid synthesis (*PSY*, *PSD*, *Z-ISO*, and *LCY*) being coordinately activated.

Pigment content analysis confirmed these changes, with phycoerythrin (PE) being 2.70–3.68 times that of the control at 10–12 h, while phycocyanin (PC) and allophycocyanin (APC) reached 1.48 times and 1.7 times the control levels at 2 h, respectively. Chlorophyll a content peaked at 1.86 times the control at 24 h. These findings suggested that SPM enhanced photosynthetic efficiency and pigment biosynthesis, contributing to the observed promotion of conchosporangial formation.

### 2.6. SPM Enhances Carbon Assimilation by Regulating the Calvin Cycle and C_2_ Photorespiration Pathways

**Figure 5:**
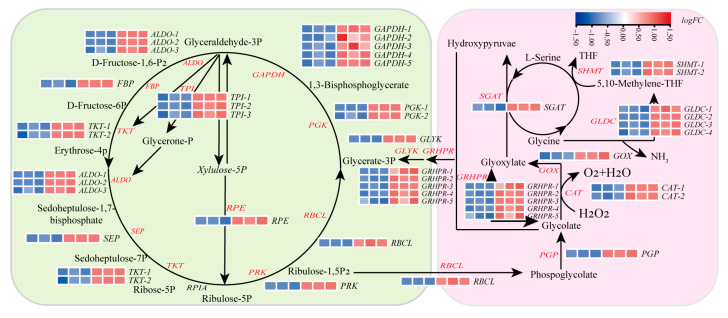
Effect of SPM on carbon assimilation in *P. haitanensis* conchosporangia. Transcriptome expression profiles of the *P. haitanensis* Calvin cycle and photorespiratory C_2_ cycle following SPM treatment. Gene names highlighted in red indicate significantly DEGs (|log_2_FC| > 1, adjusted *p*-value < 0.05), while black fonts represent non-significant genes. Conchocelis were subjected to maturation conditions and treated with 1 μM SPM for 24 h.

Transcriptomic analysis of *P. haitanensis* conchocelis treated with 1 μM SPM for 24 h revealed significant upregulation of genes associated with both the Calvin cycle and C_2_ photorespiration pathways, pivotal processes for carbon assimilation during conchosporangial formation (Figure 5). In the Calvin cycle, 25 DEGs involved in carbon fixation were identified. Notably, key genes in the carboxylation and reduction phases, including ribulose-bisphosphate carboxylase (*RBCL*), phosphoglycerate kinase (*PGK*), and glyceraldehyde-3-phosphate dehydrogenase (*GAPDH*), were significantly upregulated (*p* < 0.05), suggesting enhanced carbon fixation efficiency.

Concurrently, SPM treatment upregulated genes associated with the C_2_ photorespiration pathway (which mediates glyoxylate metabolism), including glycolate oxidase (*GOX*), serine-glyoxylate aminotransferase (*SGAT*), and glyoxylate/hydroxypyruvate reductase (*GRHPR*). In contrast, transcripts of key tricarboxylic acid (TCA) cycle genes (e.g., aconitase (*ACO*), ATP-citrate lyase (*ACLY*), and malate dehydrogenase 1 (*MDH1*)) were markedly downregulated (Figure S7). This coordinated transcriptional shift suggested that enhanced C_2_ pathway activity might functionally complement suppressed mitochondrial respiration, potentially optimizing carbon flux under SPM influence.

### 2.7. SPM Promotes Peroxisomal Biogenesis and Modulates Antioxidant Systems in P. haitanensis

**Figure 6:**
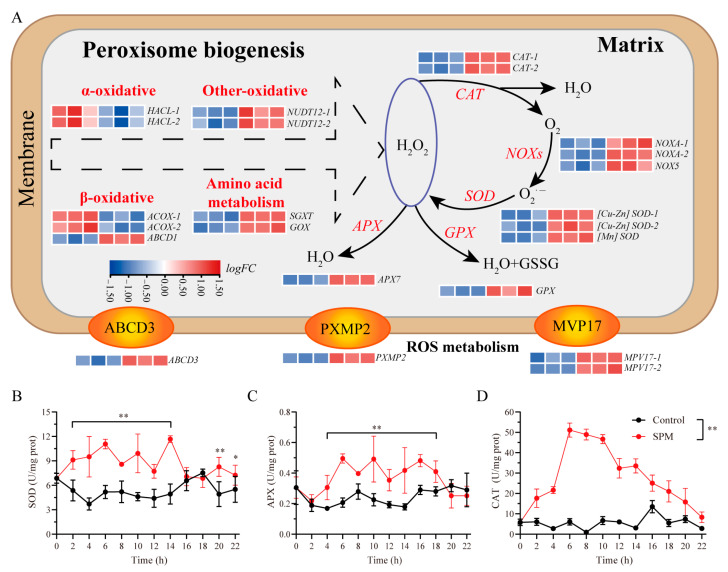
The role of SPM in peroxisome biogenesis. (**A**) Heatmap illustrating DEGs involved in peroxisome biogenesis. Transcriptional profiles of genes were recorded at 24 h. Components and gene names highlighted in red indicate significantly enriched functional categories (e.g., amino acid metabolism) or DEGs (|log_2_FC| > 1, adjusted *p*-value < 0.05). (**B**–**D**) SOD, APX, and CAT enzyme activity associated with H_2_O_2_ metabolism. Conchocelis were subjected to maturation conditions and treated with 1 μM SPM. Enzyme activity was assessed at different time points. Statistical significance was calculated by one-way ANOVA. * *p* < 0.05 and ** *p* < 0.01, compared to the control group (n = 3).

To investigate the role of SPM in managing oxidative stress during conchosporangial formation, the expression of genes related to peroxisomal biogenesis and antioxidant defense mechanisms was analyzed (Figure 6). Genes critical for peroxisomal membrane protein transport, including ATP-binding cassette subfamily D member 3 (*abcD3*) and peroxisomal membrane protein 2 (*pxmp2*), were significantly upregulated (*p* < 0.05). Additionally, enzymes involved in photorespiration, such as *GOX* and *SGAT*, showed increased expression, further supporting the role of peroxisomes in ROS production under SPM treatment.

Transcriptomic data revealed a marked upregulation of genes associated with both enzymatic and non-enzymatic antioxidant defenses in response to SPM. Key antioxidant enzymes, including *SOD*, *CAT*, *APX*, and glutathione peroxidase (*GPX*), displayed significantly elevated expression levels (*p* < 0.05).

The enzyme activity profiles mirrored these transcriptional changes. During conchosporangial formation under 1 μM SPM treatment, activities of SOD, APX, and CAT remained elevated over the 24 h period. SOD activity began to increase at 2 h, stabilizing at 7.72–11.67 U/mg protein before returning to baseline at 16–18 h, followed by a second peak at 20–22 h (*p* < 0.05). APX activity rose at 4 h, maintaining a range of 0.31–0.49 U/mg protein (*p* < 0.05) before gradually declining after 18 h. Similarly, CAT activity peaked at 6 h (51.14 ± 3.48 U/mg protein) and then progressively decreased to 8.30 ± 2.63 U/mg protein at 22 h (*p* < 0.05; Figure 6B–D).

These findings highlighted SPM’s dual role in promoting peroxisomal biogenesis and bolstering antioxidant systems, thereby managing ROS levels to support the formation of conchosporangia in *P. haitanensis*.

### 2.8. SPM-Mediated Regulation of Polyamine Metabolism in P. haitanensis Conchosporangia

**Figure 7:**
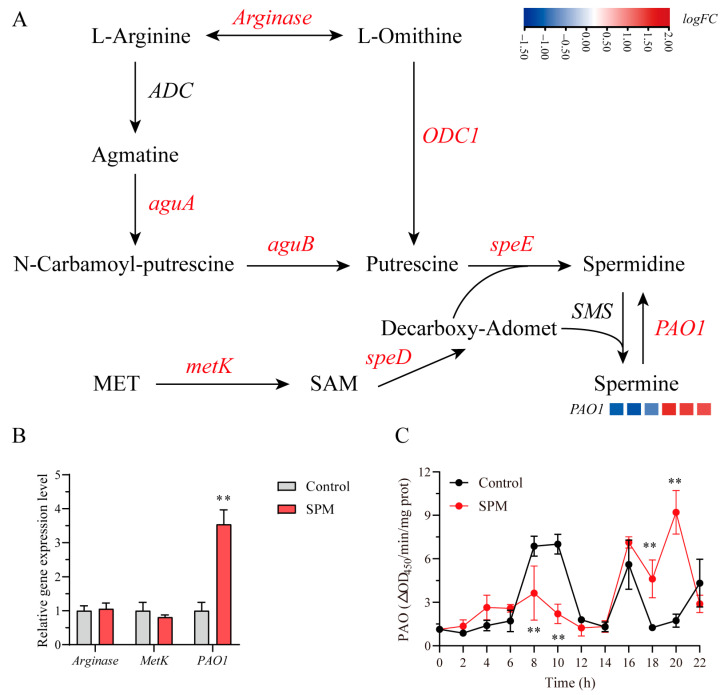
The role of SPM in the regulation of polyamine metabolism in *P. haitanensis*. (**A**) Schematic diagram of polyamine biosynthesis and catabolism pathways in *P. haitanensis*. The heatmap displays log_2_FC values compared to the control. Gene names highlighted in red represent annotated genes in the transcriptome, while those in black indicate unannotated genes. (**B**) RT-qPCR analysis of *Arginase*, *metK*, and *PAO1* gene expression after 24 h treatment with 1 μM SPM. (**C**) Time-course analysis of PAO enzyme activity following 1 μM SPM treatment. Conchocelis were cultured under maturation conditions and sampled at the indicated time points. Data represent mean ± SD (n = 3). Statistical significance was determined by one-way ANOVA (** *p* < 0.01 vs. control).

To systematically dissect the regulatory effects of SPM on polyamine metabolism in *P. haitanensis* conchosporangia, we employed an integrative approach combining transcriptome profiling, qPCR validation, and enzymatic assays. Transcriptomic analysis revealed that the majority of genes involved in polyamine biosynthesis and catabolism, such as *arginase* and *metK*, remained transcriptionally unchanged (|logFC| < 1, *p* > 0.05), whereas polyamine oxidase 1 (*PAO1*) was selectively upregulated (logFC = 1.482, *p* < 0.001; Figure 7A, Supplementary Table S2), a result further confirmed by qPCR (Figure 7B). Enzyme activity measurements revealed a biphasic PAO1 response to SPM exposure, characterized by an initial suppression at 8 h (*p* < 0.01) followed by a pronounced activation at 22 h (9.21 ± 1.45 ΔOD_450_/min/mg protein) (Figure 7C). Collectively, these findings suggested that SPM preferentially promoted its own back-conversion to SPD via PAO1 induction, while exerting minimal influence on upstream biosynthetic pathways.

### 2.9. SPM-Induced Metabolic Reprogramming Sustains Long-Term Biomass Accumulation

Building upon the SPM-induced transcriptional activation of photosynthesis, carbon assimilation, and antioxidant systems, we assessed the long-term physiological outcomes of this metabolic reprogramming. Treatment with high SPM concentrations (1 μM and 2.5 μM) significantly increased the average daily mass growth rate of conchosporangia (*p* < 0.05), achieving rates approximately 2.03 and 1.74 times higher than those of the control group. Conversely, pre-incubation with the NOX inhibitor DPI or the polyamine biosynthesis inhibitor mitoguazone (MGBG) led to a substantial reduction in the average daily mass growth rate, decreasing by 27.5% and 38.4%, respectively (Figure S8).

## 3. Discussion

This study offered valuable insights into the molecular mechanisms driving conchosporangial formation in *P. haitanensis*, with a particular focus on the role of SPM and its involvement in signaling pathways, especially those associated with ROS. Among the polyamines evaluated, SPM demonstrated the strongest promotive effect on conchosporangial formation, consistent with prior research on red algae. Previous studies have shown that polyamines like SPD and SPM enhance reproductive development in similar systems (*G. cornea* and *G. lemaneiformis*, etc.) [10,11,20,21].

The superior efficacy of SPM compared to PUT and SPD suggested that the polyamine’s structure and chain length were critical determinants of its biological activity. As a larger and more complex polyamine, SPM may engage more effectively with molecular targets [22,23], facilitating the transition from conchocelis to conchosporangia. This hypothesis is supported by findings in other algal species, where polyamines with longer chains and greater molecular complexity are often more potent in regulating developmental transitions [24]. These observations underscore the intricate relationship between polyamine structure and function in modulating reproductive processes in algae.

The most remarkable discovery in this study was the identification of O_2_·^−^ as a novel signaling molecule in SPM-mediated conchosporangial formation. Unlike earlier research in *P. haitanensis*, which has highlighted H_2_O_2_ as a key signaling molecule in this process [2], our findings revealed that O_2_·^−^ assumed a pivotal role in initiating this developmental transition. While O_2_·^−^ are well-documented signaling molecules in plant and algal growth and stress responses [25,26], their involvement in reproductive processes within marine red algae has remained elusive. Our data demonstrated that the application of the NOX inhibitor DPI markedly reduced both O_2_·^−^ production and conchosporangial formation, confirming that NOX-driven O_2_·^−^ generation was integral to SPM-induced conchosporangial development. This finding aligned with studies in higher plants, where NOX-derived O_2_·^−^ has been implicated in regulating crucial developmental events, including seed germination, root elongation, and floral induction [27,28,29]. Additionally, the temporal dynamics of O_2_·^−^ production observed in our study, characterized by two distinct peaks at 8 and 16 h, suggested that O_2_·^−^ functioned as a transient signal, orchestrating key events during the developmental progression.

Our study also provided compelling evidence that SPM enhanced photosynthetic efficiency and carbon assimilation in *P. haitanensis*. Transcriptomic analysis revealed the significant upregulation of genes associated with the light reactions of photosynthesis, including those encoding components of photosystem I (*psaO*) and photosystem II (*psbA*, *psbM*, *psbO*, *psbP*, *psbQ*, *psbS*, and *psbU*), as well as genes involved in electron transport and ATP synthesis, such as *petC* and *atpF*. These results were consistent with previous studies demonstrating the role of polyamines in modulating photosynthesis, particularly under stress conditions [30,31,32]. Polyamines like SPM are known to stabilize chloroplast membranes by binding to thylakoid structures, thereby enhancing the efficiency of light absorption and electron transport [33,34].

In the context of conchosporangial formation, enhanced photosynthetic activity likely fulfills the energy demands of this metabolically intensive developmental process [35], particularly under suboptimal environmental conditions. The upregulation of genes (including *LHCA*, *apc*, *cpc* and *hemA*, etc.) and increased pigment levels demonstrate improved light-harvesting capacity, ensuring efficient light utilization. Additionally, the increased expression of carotenoid biosynthesis genes, including *PSY* and *LCY*, underscored a protective mechanism by which carotenoids mitigate oxidative stress during this critical developmental stage [36,37]. Since conchosporangial formation frequently occurs under environmental stress, these enhancements in photosynthetic efficiency and photoprotection are likely vital for sustaining the metabolic requirements of this process.

These findings suggested that SPM functioned not only as a signaling molecule but also as a modulator of photosynthetic efficiency, providing the energy and reducing power essential for conchosporangial development. Future research could investigate the interplay between SPM-mediated ROS signaling and the regulation of photosynthetic pathways, offering deeper insights into the molecular mechanisms governing algal reproduction.

Our findings revealed that SPM treatment significantly upregulated genes involved in both the Calvin cycle and the C_2_ photorespiration pathways, underscoring its role in enhancing carbon assimilation. Specifically, key enzymes such as *RBCL*, *PGK*, and *GAPDH* were markedly upregulated, indicating elevated carbon fixation activity. These results are aligned with prior studies demonstrating that polyamines enhance carbon fixation across various plant species, including algae [38,39]. By improving Calvin cycle efficiency, SPM ensures that *P. haitanensis* can effectively incorporate carbon into organic compounds, providing both the energy and reducing power necessary for the developmental transition from conchocelis to conchosporangia.

Beyond the Calvin cycle, SPM treatment also upregulated genes involved in the C_2_ photorespiration pathway, such as *GOX*, *SGAT*, and *GRHPR*, highlighting increased photorespiratory activity. The C_2_ cycle mitigated oxidative stress associated with photosynthesis by processing glycolate and glyoxylate from RuBisCO-mediated oxygenation reactions [40]. Meanwhile, the concurrent downregulation of TCA cycle genes (*ACO*, *ACLY*, and *MDH1*) implied a redirection of mitochondrial carbon flux toward the photorespiratory pathway. This metabolic reprogramming not only facilitated carbon fixation but also contributed to the maintenance of redox homeostasis during conchosporangial development, particularly under photorespiratory stress. Taken together, these findings suggested that SPM enhanced carbon assimilation by coordinately activating both the Calvin cycle and the C_2_ pathway, thereby securing critical metabolic resources for conchosporangial formation, likely via energy redistribution and stress-responsive signaling mechanisms.

To mitigate the NOX-derived O_2_·^−^ bursts, SPM concurrently stimulated peroxisome biogenesis and antioxidant defenses, establishing a tightly regulated ROS homeostasis cycle. The significant upregulation of peroxisomal membrane proteins such as *abcD3* and *pxmp2*, along with enzymes involved in photorespiration, pointed to an active role for peroxisomes in ROS production, including H_2_O_2_. These findings were consistent with research in plants, where peroxisomes are central to ROS generation and oxidative stress regulation [41,42]. Interestingly, despite the increased peroxisomal activity, H_2_O_2_ levels remained stable following SPM treatment, suggesting the presence of an efficient antioxidant system. Key antioxidant enzymes, including SOD, APX, and CAT, as well as genes encoding *CAT*, *APX*, and *GPX*, were significantly upregulated, playing a vital role in scavenging excess ROS.

This robust antioxidant response is crucial, as oxidative stress often accompanies heightened photosynthetic activity [43]. In *P. haitanensis*, the antioxidant system appears to operate in a highly coordinated manner, selectively regulating ROS levels. The differential control of ROS suggests that SPM orchestrates redox balance during conchosporangial formation. O_2_·^−^ might serve as signaling molecules to drive developmental processes, while H_2_O_2_ levels were tightly regulated to prevent cellular damage. This selective modulation aligned with findings in plants, where polyamines modulate antioxidant enzyme activity to safeguard cells from oxidative damage while enabling ROS to function as signaling molecules [44,45]. The intricate regulation of O_2_·^−^ and H_2_O_2_ highlighted the complex interplay between oxidative stress management and developmental signaling in *P. haitanensis*, showcasing SPM’s critical role in this process. This context-dependent modulation of ROS aligned with the stress-development crosstalk model proposed by Liu et al., albeit here repurposed to regulate reproductive transitions [46].

Polyamine homeostasis, pivotal to maintaining redox equilibrium, is tightly controlled. Although the majority of genes involved in polyamine biosynthesis remained transcriptionally unaltered under SPM treatment, the significant upregulation and biphasic activity pattern of *PAO1*, a key catabolic enzyme that generates H_2_O_2_ from SPM, indicated that SPM preferentially modulated polyamine levels via degradation (Figure 7). This catabolic emphasis likely fulfilled dual functions: preserving intracellular polyamine reserves under stress while enabling metabolic adaptability essential for developmental transitions, a mechanism reminiscent of phytohormone-regulated ROS signaling observed in higher plants [47].

Interestingly, in addition to the polyamines (PUT, SPD, and SPM) identified in this study, previous research has shown that ACC and methyl jasmonate also promote conchosporangial formation in *P. haitanensis* [2]. These findings suggest that a variety of substances can induce this developmental event. Despite the widespread presence of phytohormones in *Pyropia*, their functional roles may not be as precisely differentiated as those in higher plants, likely due to the relatively primitive evolutionary status of *Pyropia*. Instead, these phytohormones may act primarily as elicitors. Notably, while multiple phytohormones can trigger conchosporangial formation through ROS signaling, they appear to utilize distinct ROS. The mechanisms underlying this specificity remain an intriguing avenue for future research.

## 4. Materials and Methods

### 4.1. Experimental Materials

Preparation of Free-Living Conchocelis: The free-living conchocelis of *P. haitanensis* (ZD-2 strain) were obtained and conserved by the Key Laboratory of Marine Biotechnology, Zhejiang Province, China. The cultivation of conchocelis and the induction of conchosporangial formation were conducted following the method described by Niu et al. (2024) [2].

Preparation of Shell-Living Conchocelis: Segments of free-living conchocelis, measuring 200–300 μm, were sprayed onto shell surfaces at a transplantation density of 100 mg/m^2^. These shells were initially kept in low-light conditions for 3 days. Following this, they were exposed to light and maintained at a constant temperature of 20 °C with a photosynthetic photon flux density (PPFD) of 15 μmol/(m^2^·s) under a photoperiod of 12 h of light and 12 h of darkness for two additional weeks. After this period, excess conchocelis filaments were carefully brushed off, the culture medium was replaced, and the light intensity was increased to 30 μmol/(m^2^·s). Once the conchocelis adequately covered the shell surfaces, the experiments to induce conchosporangial formation commenced.

### 4.2. Conchosporangia Formation Ratio Detection

Free-living conchocelis were cultivated in unsealed, transparent small glass bottles (15 mL capacity, 10 mL working volume) under various treatments, including 1 μM PUT, SPD, and SPM (Sigma-Aldrich, St. Louis, MO, USA) for 10 weeks; 0.2, 1, and 2.5 μM SPM for 8 weeks; and 1 μM SPM alone or combined with 0.1 μM DPI for 6 weeks. The cultures were maintained at 29 ± 0.5 °C with a light intensity of 20 μmol/(m^2^·s) under a photoperiod of 8 h light and 16 h darkness. The medium, supplemented with K_2_HPO_4_ to a final concentration of 110 nmol/L, was refreshed weekly during the conchosporangial maturation phase. The conchocelis and conchosporangia were monitored weekly under a microscope (Nikon Eclipse 80i, Tokyo, Japan) to assess their developmental progression. The conchosporangial formation ratio was determined using the following formula:

Percentage conchosporangia (%) = Number of conchosporangia/Total conchocelis × 100%.

Shell-living conchocelis were treated with final concentrations of 0.2, 1, and 2.5 μM SPM for a duration of 6 weeks (n = 6). Weekly, the shells were carefully removed and examined using a stereo microscope (Nikon, DS-Ri2, Tokyo, Japan). For each observation, 10 random fields of view were selected and imaged at 135× magnification. The captured images were processed and analyzed using ImageJ software (v.1.4.3.67) to quantify the proportion of conchosporangia branch formation. The proportion of conchosporangial branch formation was calculated using the following formula:

Percentage conchosporangia (%) = Conchosporangia area/(Conchocelis area + Conchosporangia area) × 100%.

### 4.3. Relative Mass Growth Rate

Free-living conchocelis cultures were maintained for 6 weeks under various treatments, including final concentrations of 0.2, 1, and 2.5 μM SPM, as well as combinations of 1 μM SPM with either 0.1 μM DPI (MedChemExpress LLC, Monmouth Junction, NJ, USA) or 0.1 μM MGBG (MedChemExpress LLC, Monmouth Junction, NJ, USA). Each treatment group was established in triplicate, with an initial fresh weight of 0.03 g/L for the conchocelis. The culture medium was partially renewed every 7 days, with half the volume replaced during each change. Upon completing the 6-week experiment, the relative mass growth rate was determined using the following formula:

Relative mass growth rate (%/day) = [Ln (W_t_/W_0_)/42] × 100%

where W_0_ is the initial FW (g), and W_t_ is the FW at 6 weeks (g).

### 4.4. Detection of H_2_O_2_, O_2_·^−^ Contents, and Related Enzyme Activities

Free-living conchocelis were cultivated with a final concentration of 1 μM SPM for 24 h, and fresh samples weighing 0.03 g were collected at 2 h intervals. The collected samples were homogenized in a cell lysis buffer (Beyotime Biotechnology, Shanghai, China) for 10 min to prepare a 10% homogenate. After centrifugation at 2500× *g* for 10 min at 4 °C, the supernatants containing enzymes, H_2_O_2_, and O_2_·^−^ were collected for further analysis. The levels of H_2_O_2_, SOD (EC 1.15.1.1), CAT (EC 1.11.1.6), and APX (EC 1.11.1.11) were quantified using assay kits provided by Nanjing Jiancheng Bioengineering Institute (Nanjing, China), while O_2_·^−^, NOX (EC 1.6.3.1), and PAO (EC 1.5.3.11) were assessed using assay kits from Suzhou Grace Biotechnology Co., Ltd. (Suzhou, China).

For cellular O_2_·^−^ detection, conchocelis were treated with 1 μM SPM or a combination of 1 μM SPM and 0.1 μM DPI for 8 h. The samples were then rinsed three times with sterile seawater. Subsequently, 0.1 μM lucigenin (MedChemExpress LLC, Monmouth Junction, NJ, USA) was added, and the samples were incubated for an additional 30 min. Fluorescence microscopy was employed for imaging, with an excitation wavelength of λex = 405 nm and an emission wavelength of λem = 492 nm.

### 4.5. Determination of Endogenous PUT, SPD, and SPM Levels in P. haitanensis

Standard solutions of PUT, SPD, and SPM (2 mg/mL in ultrapure water) were derivatized with benzoyl chloride in 2 M NaOH at 37 °C for 20 min, followed by extraction with and vacuum concentration. The resulting derivatives were reconstituted in methanol, filtered through a 0.22 μm membrane, and serially diluted (0.5–8 μg/mL) to generate calibration curves. For sample preparation, 10 mg of lyophilized powder was extracted with 5% perchloric acid for 1 h in an ice bath and centrifuged at 15,000× *g* for 30 min, and the supernatant was derivatized using the same protocol. Chromatographic analysis was performed using UPLC equipped with a Waters ACQUITY HSS T3 column (Milford, MA, USA) (1.8 μm, 30 °C), employing isocratic elution with acetonitrile:water (44:56, *v/v*) at a flow rate of 0.45 mL/min and detection at 220–230 nm. Data acquisition and quantification were conducted using MassLynx software (v.4.2), and results were exported to Excel for statistical analysis. All measurements represent the mean ± SD from three biological replicates.

### 4.6. Phycobiliprotein and Chlorophyll a Quantification

Phycobiliproteins (PE, PC, APC) were extracted from 0.02 g algal samples using PBS buffer after liquid nitrogen grinding and freeze–thaw cycles. Absorbance was measured at 565 nm, 615 nm, and 650 nm, with concentrations calculated as follows:

C_PE_ (mg/g) = (0.123A_565_ − 0.068A_615_ + 0.015A_650_) × V/(1000 × FW)

C_PC_ (mg/g) = (0.162A_615_ − 0.001A_565_ + 0.098A_650_) × V/(1000 × FW)

C_APC_ (mg/g) = (0.171A_650_ − 0.006A_565_ + 0.004A_615_) × V/(1000 × FW)

Chlorophyll *a* was extracted in 5 mL absolute methanol (24 h, 4 °C in darkness), with absorbance measured at 665 nm, 750 nm, and 652 nm. Pigment contents were determined using:

Chl *a* (mg/g) = [16.29(A_650_ − A_750_) − 8.54(A_652_ − A_750_)] × V/(1000 × FW)

where V is extract volume (mL) and FW is fresh weight (g) [48].

### 4.7. RNA Isolation, Transcriptome Sequencing, and Analysis

Approximately 100 mg of *P. haitanensis* tissue from each group was collected, snap-frozen in liquid nitrogen, and ground into a fine powder. Total RNA was extracted using the Plant RNA Isolation Kit (Meiji Bio, Guangzhou, China). The integrity of the RNA was evaluated via agarose gel electrophoresis, and its concentration and purity were determined using a NanoDrop™ One spectrophotometer (Wilmington, DE, USA). Only RNA samples meeting stringent quality standards were used for subsequent analyses.

Transcriptome sequencing was performed on the Illumina HiSeq platform at Wuhan Feisha Gene Information Co., Ltd. (Wuhan, China). After removing low-quality reads, the clean reads were aligned to the *P. haitanensis* reference genome using HISAT2 (v.2.2.1). Further mapping to the reference transcriptome was conducted with Bowtie2 software (v.2.5.1), and transcript quantification was carried out using RSEM. DEGs were identified through DESeq2 (v.1.22.2), with the criteria of |log_2_FC| > 1 and a false discovery rate (FDR) significance threshold (padj) < 0.05.

Functional annotation and enrichment analysis of the transcriptome were performed using the KEGG database (www.KEGG.jp). Enriched pathways were identified via hypergeometric testing, with pathway enrichment analysis carried out using custom scripts in R (v3.3.2, www.r-project.org). The Benjamini–Hochberg (BH) correction was applied to control the FDR.

To visualize transcriptome data, fuzzy C-means clustering was performed using the TCseq and Cairo packages in R. Heatmaps were generated using TBtools (https://github.com/CJ-Chen/TBtools-II/releases; accessed on 28 July 2023), while KEGG bubble plots were created with Oebiotech’s online platform (https://cloud.oebiotech.com/task/; accessed on 25 June 2024). Bar charts were generated using GraphPad Prism (v.8.0.2), and pathway diagrams were illustrated with Adobe Illustrator (v.2021).

### 4.8. Quantitative Real-Time PCR (qRT-PCR) Analysis

Total RNA was extracted from samples of each group using the Plant RNA Extraction Kit (Magen, Guangzhou, China). Single-stranded cDNA was synthesized using the PrimeScript™ RT Master Mix (TaKaRa Biotechnology Co., Ltd., Dalian, China), following the manufacturer’s protocol. qRT-PCR was conducted on a LightCycler^®^ 96 Real-Time PCR System (Roche, Basel, Switzerland). The primer sequences used in this study are detailed in Supplementary Table S3. The amplification protocol consisted of an initial denaturation at 94 °C for 30 s, followed by 40 cycles of 94 °C for 5 s, 60 °C for 15 s, and 72 °C for 10 s. Relative gene expression levels were calculated using the 2^−ΔΔCt^ method, with β-actin serving as the internal reference gene.

### 4.9. Statistical Analysis

All experiments were conducted in triplicate, with results expressed as the mean ± SD. Statistical analyses were performed using SPSS software (version 22.0; SPSS Inc., Chicago, IL, USA). Differences among treatment groups were evaluated using one-way ANOVA, followed by Tukey’s HSD test for multiple comparisons. A *p*-value of <0.05 was considered statistically significant.

## 5. Conclusions

This study underscored the multifaceted role of SPM in regulating conchosporangial formation in *P. haitanensis*. SPM functioned not only as a signaling molecule that stimulates ROS production but also as a modulator of photosynthetic efficiency, carbon assimilation, and antioxidant defenses. The interplay between SPM-induced ROS signaling and cellular antioxidant systems ensured a precise balance between driving developmental processes and preventing oxidative damage. These findings shed light on the molecular mechanisms underlying conchosporangial development in *P. haitanensis* and offered valuable strategies for enhancing its cultivation practices.

Moreover, this study contributed to the broader understanding of conchosporangial formation mechanisms, providing a theoretical foundation for elucidating the life cycle of algae. Further investigations are warranted to explore the crosstalk between O_2_·^−^ and other types of ROS, such as H_2_O_2_, and to identify additional signaling molecules involved in polyamine-mediated developmental transitions in algae. These efforts will deepen our understanding of algal developmental biology and may reveal novel approaches to optimize algal cultivation systems.

## Data Availability

The transcriptomic datasets analyzed in this study are publicly available in the SRA database under BioProject accession number PRJNA1203968.

## Supplementary Materials

The following supporting information can be downloaded at https://www.mdpi.com/article/10.3390/md23080309/s1: Figure S1: Contents of putrescine (PUT), spermidine (SPD), and spermine (SPM) in free-living conchocelis; Figure S2: Effect of different concentrations of polyamines on the formation of conchosporangia; Figure S3: RT-PCR validation of NOX-related gene expression in SPM-treated *P. haitanensis*; Figure S4: SPM promotes the generation of H_2_O_2_ during conchosporangial formation of *P. haitanensis*; Figure S5: Validation of the expression of randomly selected unigenes by qRT-PCR; Figure S6: Transcriptional responses of conchocelis to mature condition stimulation; Figure S7: Effect of SPM on TCA cycle-related gene expression in *P. haitanensis* conchosporangia; Figure S8: Effect of SPM on relative mass increase in *P. haitanensis* conchosporangia; Table S1: Transcriptome differential gene; Table S2: Expression profiles of polyamine metabolic genes in transcriptome analysis; Table S3: Primers for qRT-PCR.

## Abbreviations

SPM	spermine
PUT	putrescine
SPD	spermidine
O_2_·^−^	superoxide anions
H_2_O_2_	hydrogen peroxide
ACC	1-aminocyclopropane-1-carboxylic acid
DPI	diphenyliodonium chloride
MGBG	mitoguazone
SOD	superoxide dismutase
CAT	catalase
APX	ascorbate peroxidase
NOX	NADPH oxidase
PSY	15-cis-phytoene synthase
PSD	15-cis-phytoene desaturase
Z-ISO	zeta-carotene isomerase
LCY	lycopene beta-cyclase
RBCL	ribulose-bisphosphate carboxylase
PGK	phosphoglycerate kinase
GAPDH	glyceraldehyde-3-phosphate dehydrogenase
PGP	phosphoglycolate phosphatase
GOX	glycolate oxidase
SGAT	serine-glyoxylate transaminase
GRHPR	glyoxylate/hydroxypyruvate reductase
ABCD3	ATP-binding cassette subfamily D member 3
