# Supplementary material for: Spermine Promotes the Formation of Conchosporangia in Pyropia haitanensis Through Superoxide Anions
Source: Mar Drugs. 2025 Jul 30;23(8):309. doi: 10.3390/md23080309 (PMC12387882; doi:10.3390/md23080309)
Supplement: Supplementary file 1 [file marinedrugs-23-00309-s001.zip › Supplementary Figures.docx]

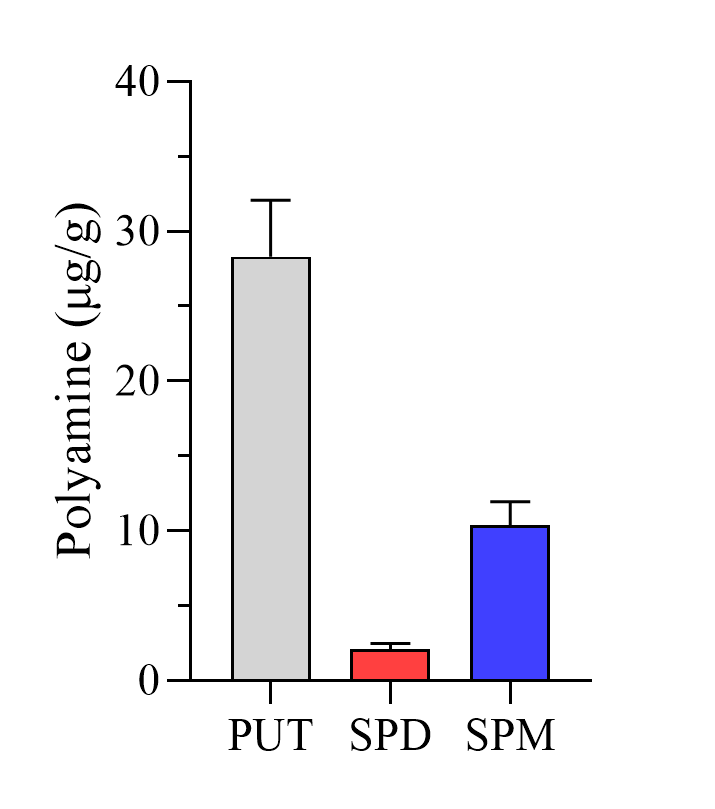


**Figure S1.** Contents of putrescine (PUT), spermidine (SPD), and spermine (SPM) in free-living conchocelis. Data are mean ± SD (n=3).


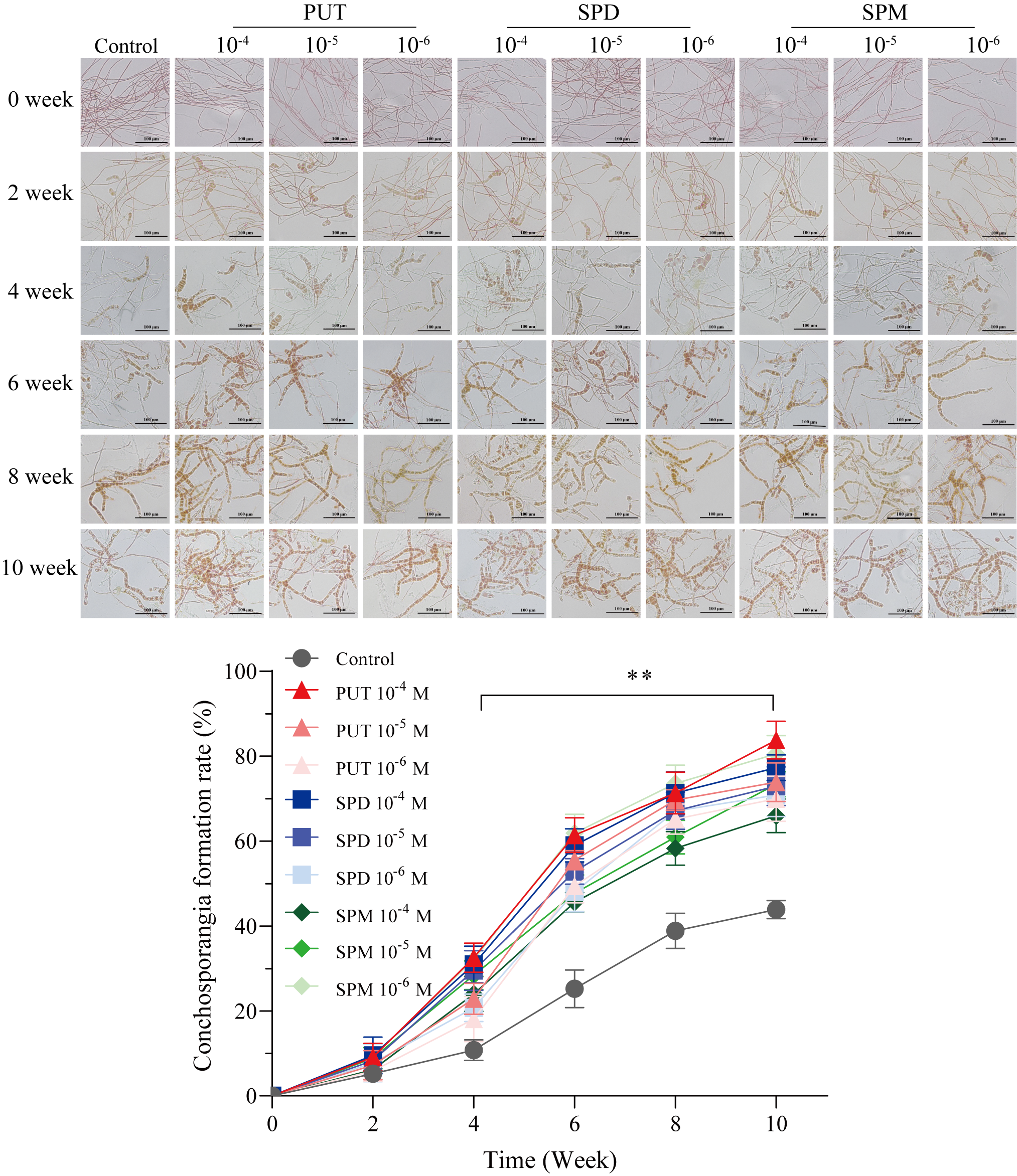


**Figure S2.** Effect of different concentrations of polyamines on the formation of conchosporangia. ***p*<0.01, compared to the control group (n = 6). Scale bar: 100 μm.

**
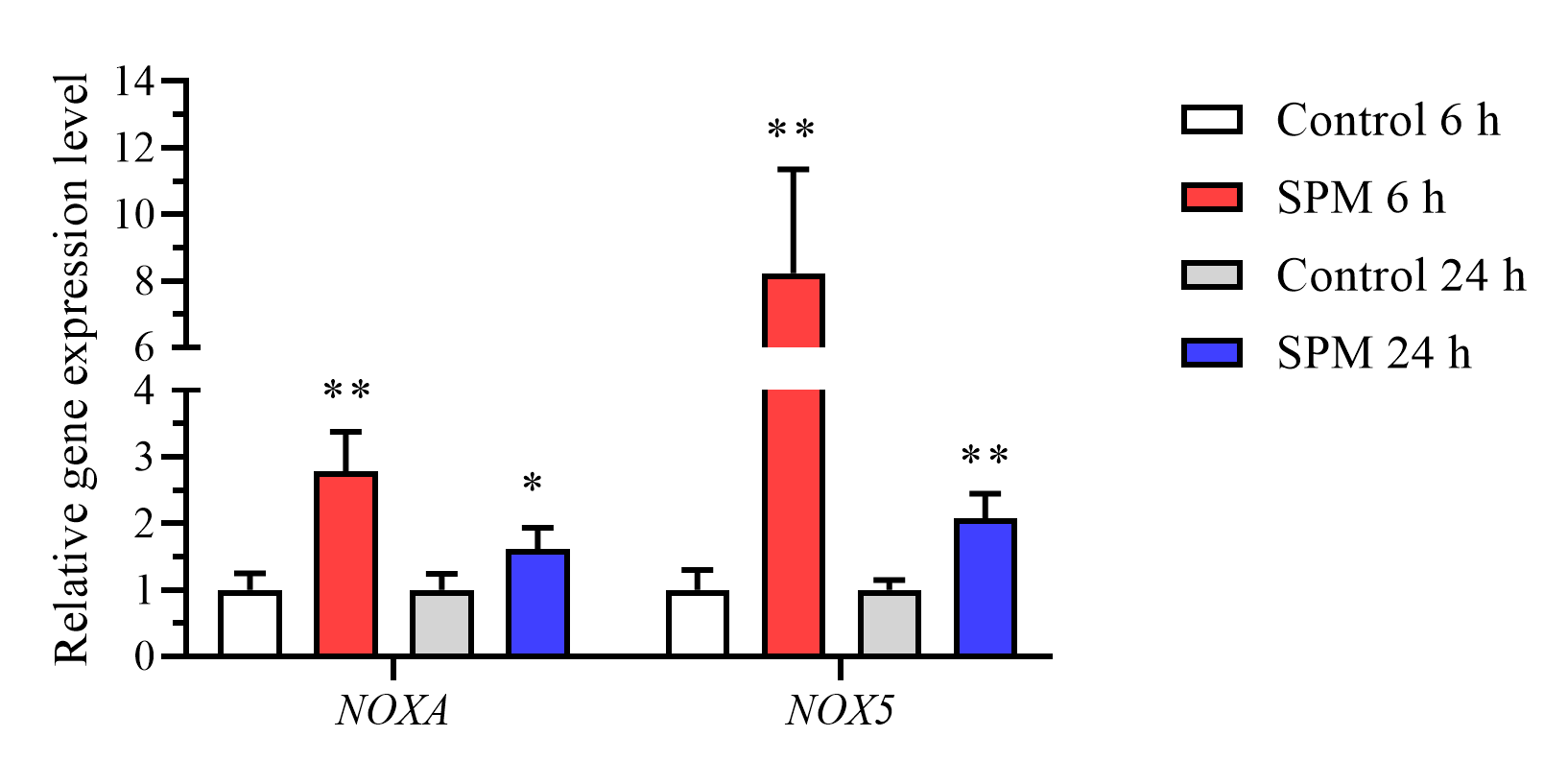
**

**Figure S3.** RT-PCR validation of NOX-related gene expression in SPM-treated *P. haitanensis.* Values represent mean ± SD (n=3). ^*^*p*<0.05, ^**^*p*<0.01 vs Control.

*
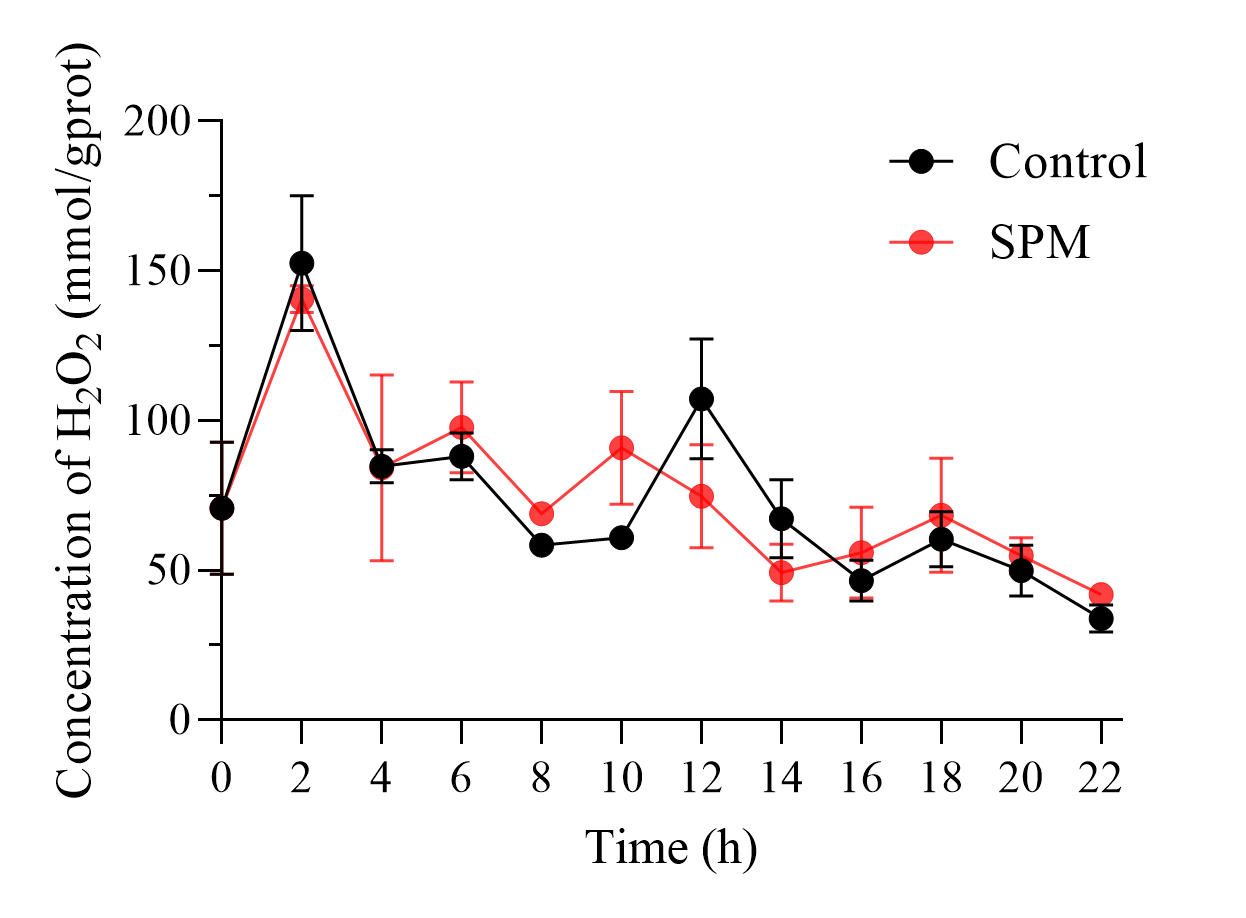
*

**Figure S4.** SPM promotes the generation of H_2_O_2_ during conchosporangial formation of *P. haitanensis*. ^*^*p*<0.05 and ^**^*p*<0.01, compared to the Control group (n = 3).

**
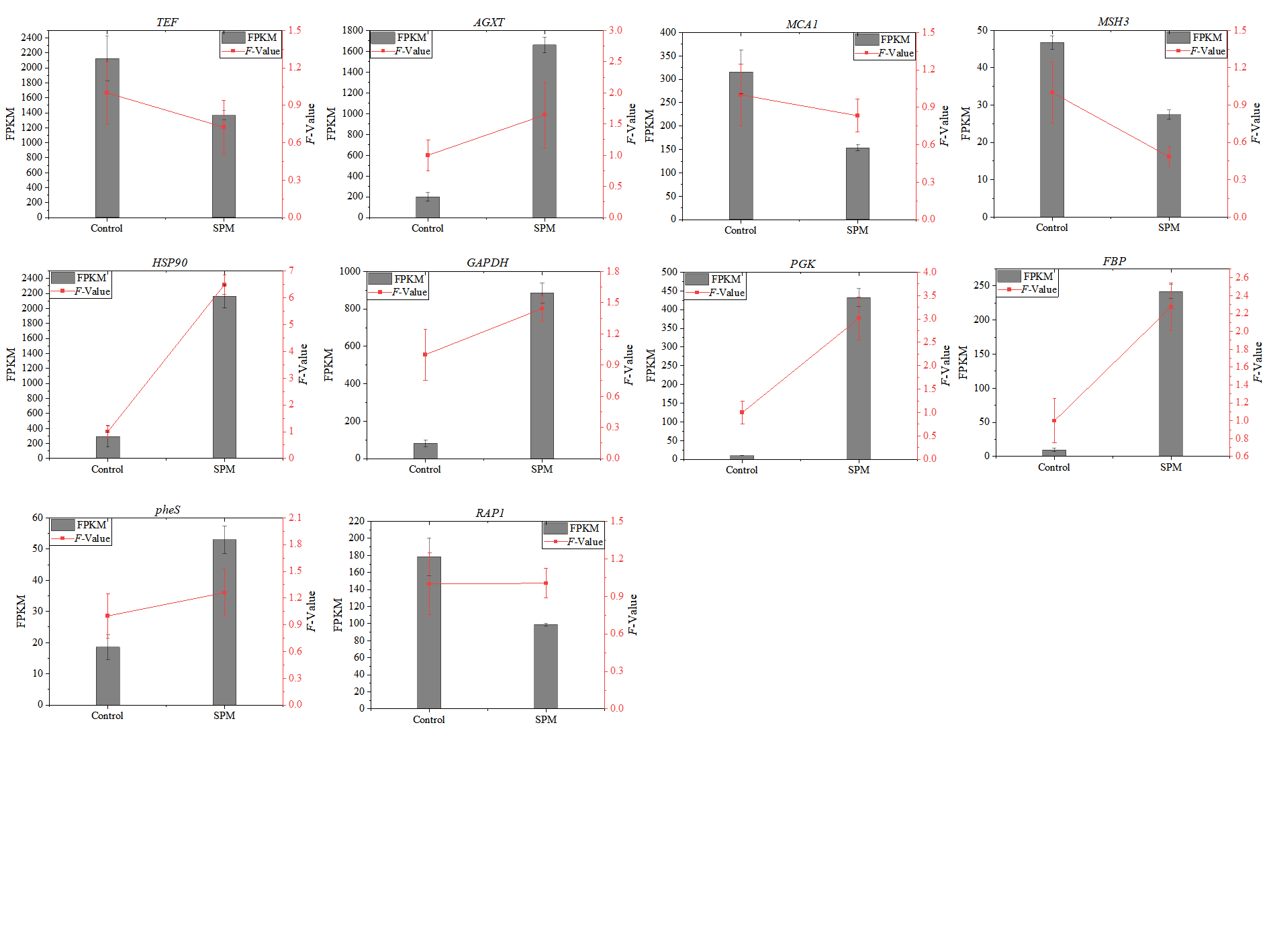
**

**Figure S5.** Validate the expression of randomly selected unigenes by qRT-PCR. (A) The gene expression at various time points within a 24-hour period under maturation conditions. (B) The gene expression of genes at different time points within a 24-hour period under maturation conditions with a 1 μM SPM treatment. Error bars indicate standard errors of the means (n = 3).


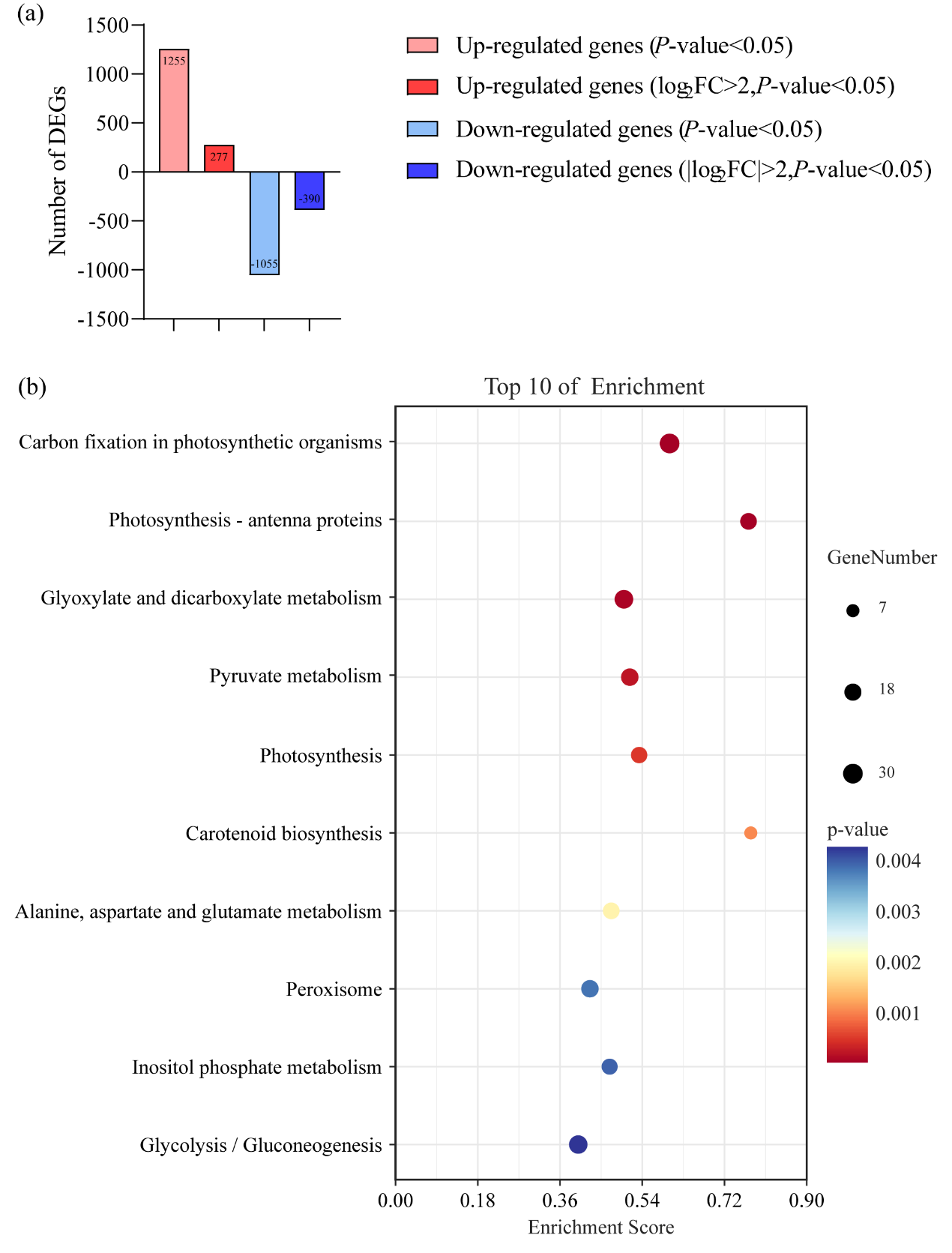


**Figure S6.** Transcriptional responses of conchocelis to mature conditions stimulation. (a) Number of differentially expressed genes (DEGs) between different groups. (b) KEGG enrichment of DEGs between contrasted groups.


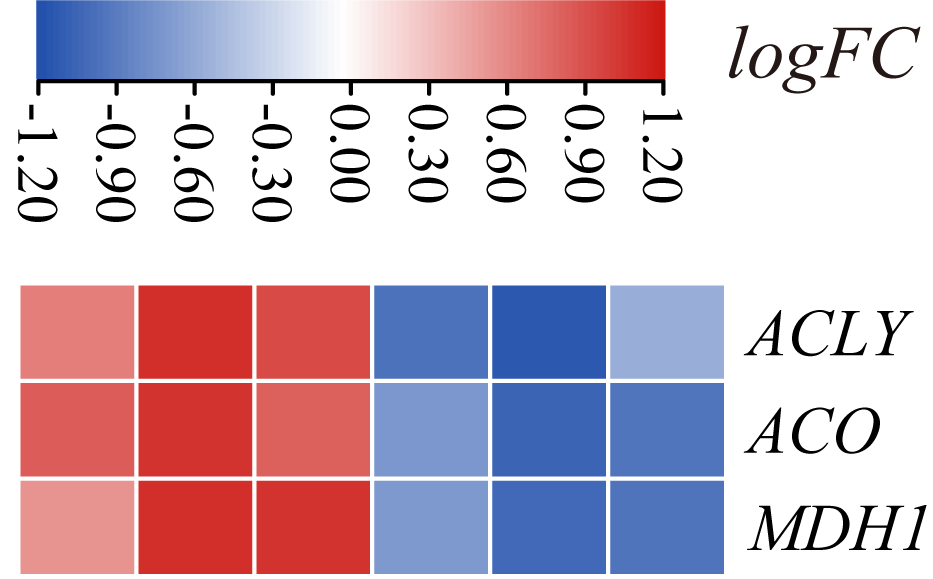


**Figure S7.** Effect of SPM on TCA cycle-related gene expression in *P. haitanensis* conchosporangia. Conchocelis were subjected to maturation conditions and treated with 1 μM SPM for 24 h.


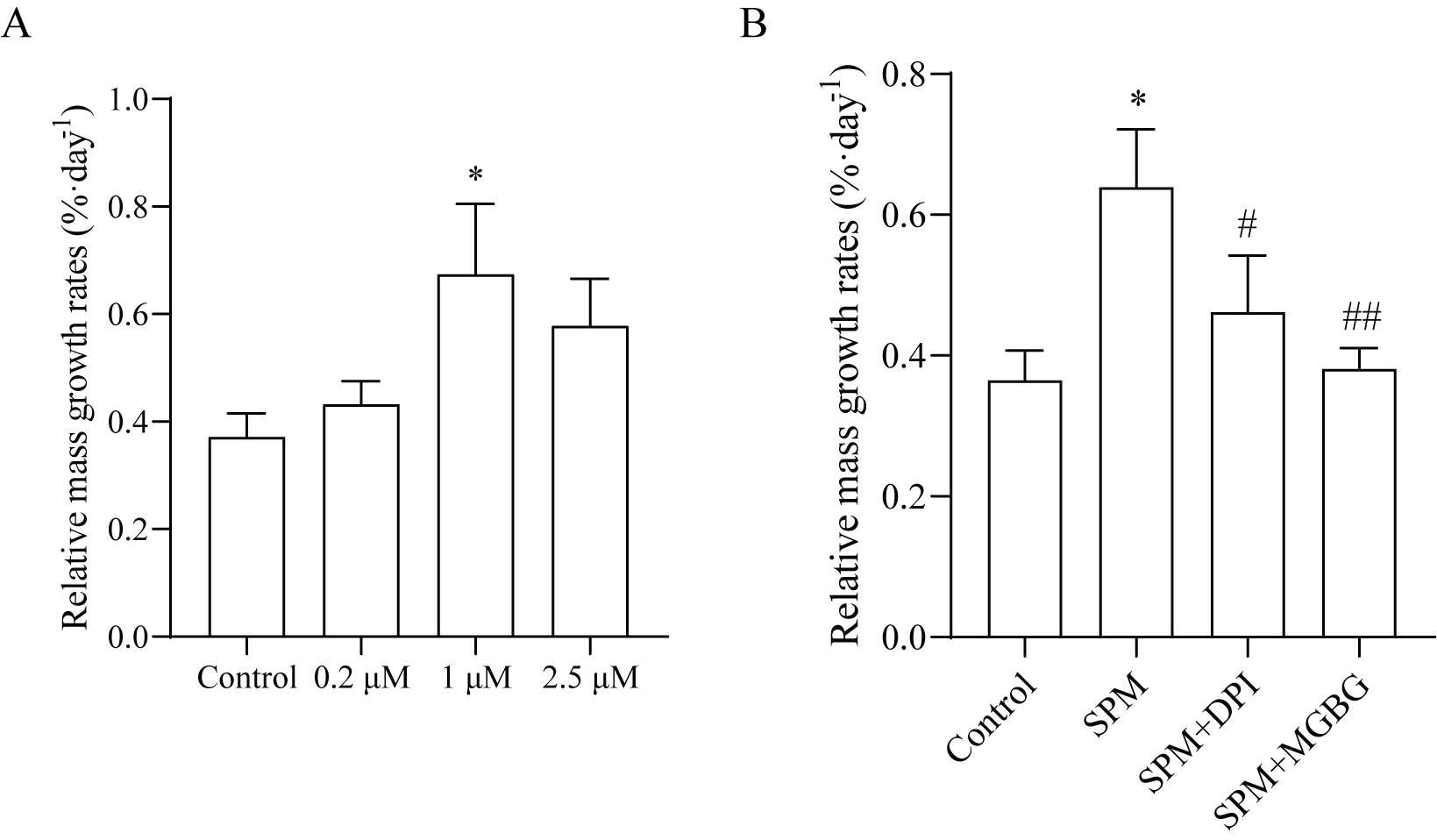


**Figure S8.** Effect of SPM on relative mass increase in *P. haitanensis* conchosporangia. (A, B) The relative mass growth rates of *P. haitanensis* conchosporangia treated with SPM or inhibitors (DPI and MGBG) over a 6-week period. Statistical significance was checked by one-way ANOVA, followed by Tukey’s HSD test. ^**^*p*<0.05, compared to the control group (n = 3); ^#^*p*<0.05 and ^##^*p*<0.01, compared to the SPM group (n = 3).
