# Supplementary material for: Spermine Promotes the Formation of Conchosporangia in Pyropia haitanensis Through Superoxide Anions
Source: Mar Drugs. 2025 Jul 30;23(8):309. doi: 10.3390/md23080309 (PMC12387882; doi:10.3390/md23080309)
Supplement: Supplementary file 1 [file marinedrugs-23-00309-s001.zip › Supplementary Table S2-S3.docx]

**Table S2.** Expression profiles of polyamine metabolic genes in transcriptome analysis

| Gene ID | Gene symbol | LogFC | *p*-Value | FDR |
| --- | --- | --- | --- | --- |
| Pha002584 | *Arginase* | -2.096 | 0.215 | 0.326 |
| Pha003461 | *aguA* | 0.603 | 0.057 | 0.107 |
| Pha007898 | *aguB* | -0.815 | 0.000 | 0.000 |
| Pha006424 | *ODC1* | 0.983 | 0.000 | 0.000 |
| Pha005879 | *metK* | 0.405 | 0.047 | 0.092 |
| Pha000846 | *speD* | 0.446 | 0.003 | 0.007 |
| Pha007190 | *speE* | 0.517 | 0.002 | 0.005 |
| Pha009106 | *PAO1* | 1.482 | 0.000 | 0.000 |

**Table S3** Primers for qRT-PCR

| **Primers** | **Sequences（5’-3’）** | **PCR products (bp)** |
| --- | --- | --- |
| *FBP* | F: GGCAAGTGCTGCGTGATG | 149 |
|  | R: GCAAAGATGGAGCCAATCGA |  |
| *GAPDH* | F: GCTATCAGCGGCTTTGGC | 110 |
|  | R: CCGTCTTGACACCCGAGGT |  |
| *AGXT* | F: CCCGAGGGCGTTGACTC | 136 |
|  | R: CGTCGTAGCGGCTGTTGTAG |  |
| *PGK* | F: GACGTCGTTGGGAGCTACAAC | 106 |
|  | R: CCGACAGCCAGTACTTGATGC |  |
| *TEF* | F: AAGACGCCCAACCAGTACATC | 145 |
|  | R: TCTTCTCCTCATTCTGCCACA |  |
| *MCA* | F: ACGATGAAGTCCTAACCCTGG | 107 |
|  | R: TCTCGTCCAGACCATCCTCTT |  |
| *pheS* | F: TTTGACGACCTCGGCGTG | 103 |
|  | R: TGTGGGCGGTCATGTGTG |  |
| *RPA1* | F: CGACGACCAACAACACCAAC | 102 |
|  | R: TGGCAATCCGCTTCTTAGCA |  |
| *MSH3* | F: ACGTCTGCTATCTTGGAGCG | 184 |
|  | R: GGGGGTAGTGGGTGACAAAG |  |
| *HSP90* | F: CAAGACGGACAAGACGGTCA | 147 |
|  | R: TCGTCGTCGTCAATCTGCAA |  |
| *β-actin* | F: GGTGGTGATTGACAATGGGTCT | 193 |
|  | R: CAGCGGGTACTTGATGAGCAG |  |
| *metK* | F: GAAGAACTTTGACCTGCGGC | 130 |
|  | R: AAGCTTCTTGACCGTCTCCC |  |
| *Arginase* | F:GGCCTGGAAAAGTTCACCCT | 183 |
|  | R: GGCCTCTGACATCCAAGCTT |  |
| *PAO1* | F:CGACTTGGAGGTGGAGTTG | 117 |
|  | R:ACGTTGTCAAAGTCGGTGGT |  |
| *NOXA* | F:CACGCACCTCCTCAAGC | 212 |
|  | R:CGTCCTCGTCCGTTTCC |  |
| *NOX5* | F:GCCGTTTGACAAGGCGATGC | 117 |
|  | R:GCCCAGAGGGAGCCGATAAAGT |  |
